# Reevaluating anticoagulation therapy for sepsis-associated disseminated intravascular coagulation based on endothelial glycocalyx shedding quantification: a single-center, retrospective, observational pilot study

**DOI:** 10.3389/fmed.2025.1566753

**Published:** 2025-07-16

**Authors:** Keiko Suzuki, Akio Suzuki, Kazuyuki Sumi, Kodai Suzuki, Tomoaki Yoshimura, Shozo Yoshida, Nobuyuki Tetsuka, Hideshi Okada

**Affiliations:** ^1^Practical Pharmaceutical Research Center, Gifu Pharmaceutical University, Gifu, Japan; ^2^Department of Emergency and Disaster Medicine, Gifu University Graduate School of Medicine, Gifu, Japan; ^3^Department of Pharmacy, Gifu University Hospital, Gifu, Japan; ^4^Department of Infection Control, Gifu University Graduate School of Medicine, Gifu, Japan; ^5^Abuse Prevention Emergency Medicine, Gifu University Graduate School of Medicine, Gifu, Japan; ^6^Center for One Medicine Innovative Translational Research, Gifu University Institute for Advanced Study, Gifu, Japan

**Keywords:** anticoagulant, disseminated intravascular coagulation, glycocalyx, sepsis, syndecan-1

## Abstract

**Background:**

Sepsis-induced disseminated intravascular coagulation (DIC) is associated with critical conditions and linked to a high mortality rate. Anticoagulants such as recombinant human soluble thrombomodulin (rhTM) and antithrombin are used to treat sepsis-associated DIC; however, their efficacy remains controversial. Syndecan-1, a biomarker of endothelial glycocalyx injury, has been proposed as a potential indicator of sepsis severity and prognosis. This study aimed to investigate the association between serum syndecan-1 levels and recovery from sepsis-associated DIC in patients treated with anticoagulants.

**Methods:**

A retrospective observational study was conducted at Gifu University Hospital. Patients aged ≥ 20 years with sepsis-associated DIC treated with anticoagulants (rhTM and antithrombin III) for ≥2 days were included. Serum syndecan-1 levels were measured at baseline, during treatment, and at 2 days after therapy. The relationship between syndecan-1 levels and recovery from DIC, assessed using the Japanese Association for Acute Medicine (JAAM)-2 and JAAM-DIC criteria, was analyzed.

**Results:**

Thirteen patients were included. Serum syndecan-1 levels peaked at the start of anticoagulation therapy, decreased during treatment, and increased after cessation of therapy. Recovery from DIC was associated with lower post-treatment syndecan-1 levels (*p* < 0.05). In patients who did not recover, syndecan-1 levels increased by more than 30%, correlating with poor outcomes, including mortality.

**Conclusion:**

Syndecan-1 is a potential marker for monitoring endothelial injury and recovery from sepsis-associated DIC. Extended anticoagulant therapy may improve outcomes by reducing endothelial damage and potentially enhancing recovery from DIC in patients with sepsis. Further large-scale studies are required to confirm these findings.

## 1 Introduction

Sepsis is a life-threatening organ dysfunction caused by an abnormal host response to infection (1). Sepsis often results in disseminated intravascular coagulation (DIC), often due to marked coagulation activation from monocytes/macrophages, the vascular endothelium, and microthrombosis from increased tissue factor expression caused by the action of endotoxins and cytokines. DIC is a strong predictor of mortality in patients with sepsis, regardless of its severity (2). The first step in treating DIC is to treat the underlying disease. Because sepsis-associated DIC is essentially a marked activation of coagulation, anticoagulants may inhibit it; however, the need for anticoagulation therapy in sepsis-associated DIC remains controversial. This is partly due to limited and inconsistent evidence, and also because clinical attitudes toward sepsis-associated DIC differ significantly across countries. In many countries, sepsis-associated DIC is treated only for underlying diseases and is not specifically treated with anticoagulants. In the international guidelines for managing sepsis and septic shock (SSCG 2021), the reference to anticoagulation in treating sepsis-associated DIC was removed due to insufficient supporting evidence (3). Despite this, some studies have reported that anticoagulation with drugs such as heparin, antithrombin, and recombinant human soluble thrombomodulin (rhTM) may be effective in sepsis-associated DIC, although these results remain controversial (4–9). The current reliance on DIC scores for evaluating the efficacy of anticoagulation therapy for sepsis-associated DIC is limiting. Sepsis-associated DIC is typically evaluated at a single point, but the current scoring system is too unreliable to accurately determine whether the condition improves or worsens over time.

The surface of a healthy vascular endothelium is covered with a layer of proteoglycans and glycoproteins called the glycocalyx, which contributes to the regulation of vascular permeability and the inhibition of blood coagulation. The glycocalyx is injured when systemic inflammation occurs because of sepsis, resulting in increased vascular permeability and thrombus formation, leading to organ disorders. Syndecan-1, a core protein of the glycocalyx, is released into the blood when the glycocalyx disintegrates; it has been reported as a marker of glycocalyx disorders in several studies (10–12). Recent reports have shown that serum syndecan-1 levels are correlated with sepsis severity, organ dysfunction, and mortality in patients with sepsis (13–15). Additionally, in patients with DIC associated with sepsis, serum syndecan-1 has been reported to correlate with DIC score (13). These findings suggest that syndecan-1 may serve as a marker for endothelial injury and treatment response. In this study, serum syndecan-1 levels were measured in patients with sepsis-associated DIC treated with anticoagulant therapy, and the association between syndecan-1 levels and outcomes after sepsis-associated DIC treatment was investigated.

## 2 Materials and methods

### 2.1 Patients

This single-center, retrospective, observational pilot study was conducted at Gifu University Hospital (Gifu, Japan). Patients aged 20 years or older who were admitted to the intensive care unit (ICU) from March 2019 to December 2021, had serum syndecan-1 levels measured in previous studies (16, 17), were diagnosed with sepsis-associated DIC during hospitalization, and received anticoagulation therapy for at least 2 days were included. Anticoagulation therapy was administered at the discretion of the attending physician, typically when antithrombin III levels were below 70%. Patients were excluded if they had insufficient anticoagulant agent dosing, did not have blood samples collected on the second day after the end of anticoagulation therapy, or developed thrombocytopenia due to medications or other diseases during anticoagulation therapy. The causes of thrombocytopenia were determined based on clinical judgment and review of the medical records.

### 2.2 Data collection and study design

Blood samples were routinely collected every morning from eligible patients upon ICU admission, and the data from these samples were used for analysis. All laboratory data, excluding serum syndecan-1 levels and other patient characteristics, were obtained from hospital electronic medical records. Severity was assessed using the Sequential Organ Failure Assessment score (18) during sepsis-associated DIC diagnosis. The dataset for serum syndecan-1 levels was derived from data previously investigated by our group (16, 17). In previous studies, serum syndecan-1 concentrations have been measured using an enzyme immunoassay (950.640.192; Diaclone, Besançon, France).

At the time of sepsis diagnosis, the International Society on Thrombosis and Hemostasis (ISTH) criteria for sepsis-associated coagulopathy (SIC) (19, 20), ISTH overt-DIC criteria (19, 20), and the Japanese Association for Acute Medicine (JAAM) DIC criteria (1, 21), including the JAAM-2 criteria (22), were evaluated. Each of these criteria has unique strengths in terms of sensitivity, specificity, and clinical applicability. Among them, the JAAM-DIC and JAAM-2 criteria were selected as the primary diagnostic tools for evaluating recovery from sepsis-associated DIC, as they are widely used in clinical practice in Japan and allow for earlier detection compared to the other criteria. A comparative summary is provided in Supplementary Table 1. Assessments were conducted daily from the day before a clear sepsis-associated DIC diagnosis to 2 days after the end of anticoagulation therapy. Fibrin-related markers (fibrinogen degradation products [FDP] and D-dimer) were selected for the ISTH SIC and overt DIC criteria. Scoring thresholds for these markers were determined based on previous studies by Gando et al. (21). The data were retrospectively analyzed.

### 2.3 Endpoint

The primary endpoint was the association between recovery from sepsis-associated DIC based on the JAAM-2 criteria at the end of anticoagulation therapy and changes in serum syndecan-1 levels after anticoagulant therapy. The same assessment was conducted using the JAAM-DIC criteria at the end of anticoagulation therapy.

### 2.4 Statistical analysis

Descriptive statistics were calculated as medians (interquartile ranges) or proportions, as appropriate. Patients were classified into two groups based on recovery from sepsis-associated DIC according to the DIC criteria at the end of anticoagulation therapy (JAAM-2 and JAAM-DIC criteria), and increases in serum syndecan-1 levels after anticoagulation therapy were compared. Since there is no established cutoff value for serum syndecan-1 levels, evaluations were conducted at three points of increase: 10, 30% (main analysis), and 50%. These cutoff values were selected based on previous reports and to explore the possible thresholds related to prognosis. A recent publication by Qian et al. (23) also used dynamic changes in syndecan-1 to assess prognostic significance. Univariate differences between groups were evaluated using Fisher’s exact test, with *p*-values < 0.05 indicating statistical significance. Statistical analyses were performed using R version 4.4.1 (R Foundation for Statistical Computing, Vienna, Austria).

## 3 Results

**Figure 1:**
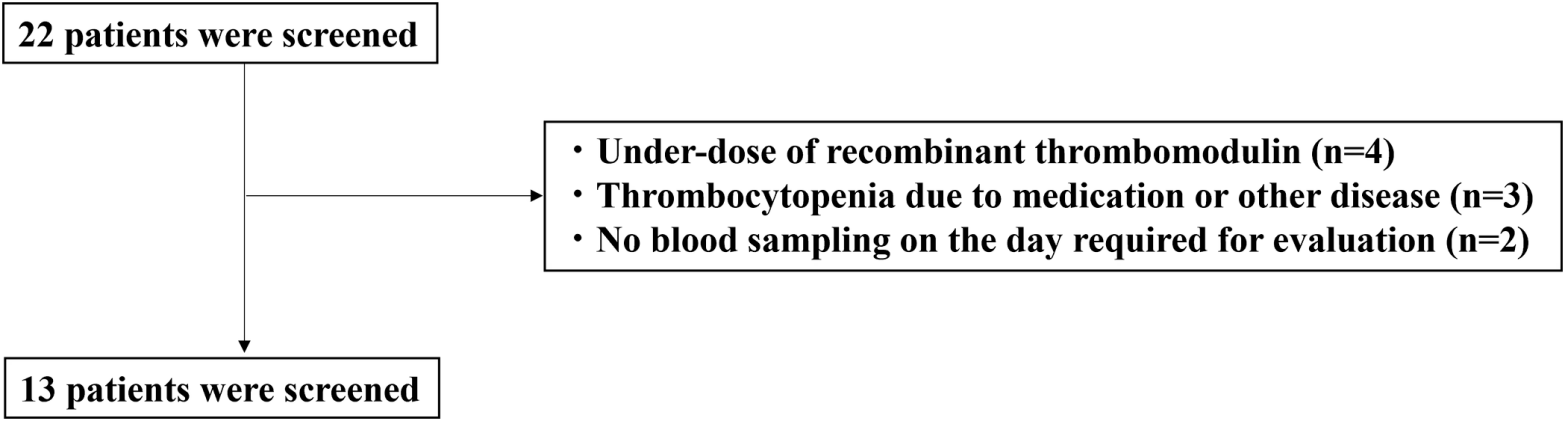
CONSORT diagram.

The characteristics of the enrolled patients are shown in Figure 1. A total of 22 patients with sepsis-associated DIC were enrolled in this study. Nine patients were excluded due to underdosing of anticoagulants (*n* = 4), thrombocytopenia due to medication or other diseases (*n* = 3), or not having blood drawn on the day required for evaluation (*n* = 2). Thirteen patients met the inclusion criteria.

The patient demographics are shown in Table 1. The median age was 71.0 years (interquartile range [IQR]: 61.0–78.0), and the median duration of hospital stay was 51.0 days (IQR: 42.0–66.0). In all 13 patients, the antithrombin III level was < 70% at the start of anticoagulant therapy, and recombinant antithrombin III was administered to 12 patients. The median duration of rhTM administration was 6 days (IQR: 5–6). All 13 patients received rhTM as anticoagulation therapy; all but one of the 12 received antithrombin, and none of the patients received heparin.

**Table 1:** Patient demographics.

Characteristics	Value
Age, years, median (IQR)	71 (61.0–78.0)
Sex, male/female, *n* (%)	11 (84.6) / 2 (15.4)
Weight, kg, median (IQR)	60.0 (54.4–70.0)
SOFA score at diagnosis of sepsis-associated DIC, median (IQR)	11.0 (9.0–12.0)
Primary disease, *n* (%)	
Burn	2 (15.4)
Heatstroke	2 (15.4)
Peritonitis	2 (15.4)
Septic shock	2 (15.4)
Trauma	2 (15.4)
Necrotizing fasciitis	3 (23.1)
Length of ICU, days, median (IQR)	27.0 (18.0–42.0)
Acute hemodiafiltration, *n* (%)	6.0 (75.0)
Ventilator, *n* (%)	13.0 (100.0)
Anticoagulant therapy, *n* (%)	
Recombinant human soluble thrombomodulin	13 (100.0)
Antithrombin	12 (92.3)
Heparin	0 (0.0)
Serum parameters at diagnosis of DIC	
AST (U/L)	34.0 (25.0–87.0)
ALT (U/L)	30.0 (18.0–50.0)
CRE (mg/dL)	1.16 (0.88–2.40)
BUN (mg/dL)	34.1 (19.6–43.7)
CRP (mg/dL)	17.2 (11.0–33.2)
WBC (×10^3^/μL)	5.59 (5.01–14.39)
PLT (×10^3^/μL)	75.0 (60.0–100.0)
FIB (mg/dL)	398.0 (308.0–694.0)
FDP (μg/mL)	18.6 (7.3–33.3)
D-dimer (μg/mL)	11.5 (2.9–23.1)
ATIII	49.0 (43.0–62.0)

All data indicate median, 25–75th percentile unless otherwise indicated. ALT, alanine aminotransferase; AST, aspartate aminotransferase; ATIII, antithrombin III; BUN, blood urea nitrogen; CRE, creatinine; CRP, C-reactive protein; DIC, disseminated intravascular coagulation; FIB, fibrinogen; FDP, fibrin degradation product; ICU, intensive care unit; IQR, interquartile range; PLT, platelets; SOFA, Sequential Organ Failure Assessment; WBC, white blood cells.

**Figure 2:**
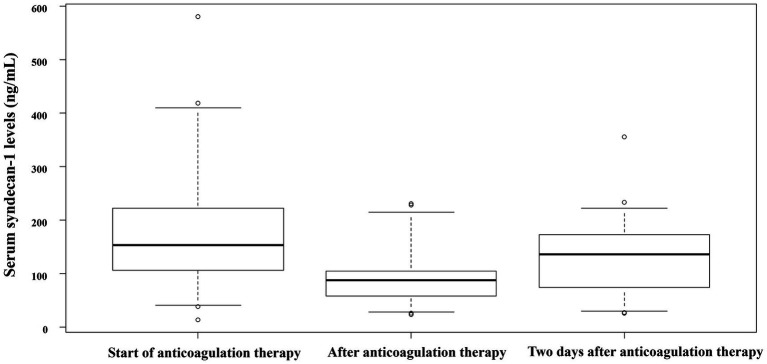
Serum syndecan-1 concentration at each time point. Serum syndecan-1 levels in patients with sepsis-associated DIC at various time points: start of anticoagulation therapy for sepsis-associated DIC, end of anticoagulation therapy for sepsis-associated DIC, and 2 days after the end of anticoagulation therapy for sepsis-associated DIC. All data indicate the median and 25th–75th percentile, unless otherwise indicated. DIC, disseminated intravascular coagulation.

The median serum syndecan-1 levels peaked at the start of anticoagulation therapy, decreased at the end of the therapy, and increased again 2 days after the completion of anticoagulation therapy (Figure 2). At the start of anticoagulant therapy, all 13 patients met the SIC score of 4 points or more, and two patients met the ISTH overt-DIC score. By 24 h after the start of anticoagulation therapy, 11 of the 13 patients had an overt DIC score. By the end of the anticoagulant therapy, all patients had recovered from the overt DIC score. Two deaths occurred during the study period.

### 3.1 Relationship between recovery from sepsis-associated DIC at the end of anticoagulant therapy and the change in serum syndecan-1 levels after treatment (JAAM-2 criteria)

By the end of anticoagulation therapy, eight of the 13 patients (61.5%) did not recover from sepsis-associated DIC. Table 2 shows the increase in serum syndecan-1 concentration at each cutoff point. Considering a 30% variation in serum syndecan-1 concentration, six out of eight patients in the non-sepsis-associated DIC recovery group showed an increase, while none of the patients in the sepsis-associated DIC recovery group exhibited an increase. There was a significant correlation between sepsis-associated DIC recovery and serum syndecan-1 concentration at the end of treatment (Table 2, *p* < 0.05). In both cases of death, the patients were unable to recover from sepsis-associated DIC at the end of anticoagulant therapy and showed an increase in serum syndecan-1 levels > 30% after treatment. Considering a 10% variation in serum syndecan-1 levels, six of eight patients in the non-recovery group had increased serum syndecan-1 levels, while only one patient in the recovery group had an increase (Table 2, *p* = 0.103).

**Table 2:** Relationship between recovery from sepsis-associated DIC at the end of anticoagulant therapy and the change in serum syndecan-1 levels after treatment (JAAM-2).

Cutoff for syndecan-1 change	Sepsis-associated DIC recovery status	Syndecan-1 increase ≥ cutoff (*n*)	Syndecan-1 increase < cutoff (*n*)	*p*-value
10%	Not Recovered (*N* = 8)	6	2	*p* = 0.103
	Recovered (*N* = 5)	1	4	
30% (Main Analysis)	Not Recovered (*N* = 8)	6	2	*p* < 0.05
	Recovered (*N* = 5)	0	5	
50%	Not Recovered (*N* = 8)	5	3	*p* = 0.075
	Recovered (*N* = 5)	0	5	

DIC, disseminated intravascular coagulation. Fisher exact test, since there is no established cutoff value for serum syndecan-1 levels, evaluations were conducted at three points of increase: 10, 30% (main analysis), and 50%.

When assessing a 50% variation in serum syndecan-1 levels, five out of eight patients in the non-sepsis-associated DIC recovery group showed an increase, whereas none of the patients in the sepsis-associated DIC recovery group exhibited an increase. A trend toward correlation was observed between sepsis-associated DIC recovery at the end of treatment and serum syndecan-1 levels (Table 2, *p* = 0.075).

### 3.2 Relationship between recovery from sepsis-associated DIC at the end of anticoagulant therapy and the change in serum syndecan-1 levels after treatment (JAAM-DIC)

We examined the recovery of sepsis-associated DIC and fluctuations in serum syndecan-1 levels according to the JAAM-DIC criteria. The results were identical to those of the JAAM-2 criteria (Supplementary Table 2). Specifically, patients who did not recover from sepsis-associated DIC exhibited a ≥ 30% increase in serum syndecan-1 levels after anticoagulant therapy, while no such increase was observed in the recovery group. This trend was similarly observed at the 10 and 50% cutoff levels, supporting the robustness of syndecan-1 as a biomarker across both diagnostic criteria.

## 4 Discussion

To our knowledge, this is the first study to examine the association between sepsis-associated DIC and the endothelial glycocalyx components after anticoagulant therapy. Serum syndecan-1, a marker of vascular endothelial glycocalyx injury, decreased after the start of anticoagulation therapy. Patients whose syndecan-1 levels remained low after therapy tended to show recovery from DIC, whereas those with increased levels tended not to recover. In addition, the two patients who died did not recover from sepsis-associated DIC at the end of anticoagulant therapy and had serum syndecan-1 levels increased by > 30% after treatment. These findings suggest persistent endothelial injury after treatment and indicate that syndecan-1 may reflect treatment response or prognosis, although causality cannot be confirmed.

### 4.1 Syndecan-1 may indicate sepsis-associated DIC status through vascular status

Syndecan-1 is a core protein of the vascular endothelial glycocalyx that is shed from the vascular endothelium and released into the blood when the glycocalyx is injured. Consequently, it is considered a marker for quantifying vascular endothelial glycocalyx injury. Previous studies have indicated that syndecan-1 is a marker for the prognosis and severity of sepsis, acute kidney injury, trauma, and heart failure (14, 16, 24), suggesting that it may serve as a predictive biomarker of mortality in critically ill patients (14, 16, 24). Sepsis-associated DIC is characterized by increased blood coagulation and is believed to be closely associated with vascular endothelial cell disorders and glycocalyx injury. Ikeda et al. further associated serum syndecan-1 levels with the onset of sepsis-associated DIC and subsequent mortality (13). In this study, patients with higher serum syndecan-1 levels after completion of sepsis-associated DIC treatment were found to have elevated DIC scores and death, suggesting that serum syndecan-1 level may be a predictive marker for sepsis-associated DIC treatment.

### 4.2 Anticoagulation therapy and syndecan-1

Treatment approaches for sepsis-associated DIC differ significantly between Japan and other countries. Anticoagulant therapies for sepsis-associated DIC include heparin, which has long been in use, as well as antithrombin and rhTM. However, other countries do not consider sepsis-associated DIC a specific treatment target (3). In Japan, the use of antithrombin and rhTM for sepsis-associated DIC is more common than in other countries, where these agents are still considered investigational. Antithrombin and rhTM, used as anticoagulants in the present study, are routinely used in clinical practice in Japan, though their use is not recommended in many countries (25). In Japan, J-SSCG 2020 (26) weakly recommends administering antithrombin and rhTM to patients with sepsis-associated DIC. The standard duration of anticoagulant therapy for sepsis-associated DIC recommended in Japan is a 6-day regimen of antithrombin III preparations and rhTM when serum antithrombin levels are < 70%. In addition, the administration of antithrombin preparations and rhTM for sepsis-associated DIC is known to have anticoagulant, anti-inflammatory, and vascular endothelial glycoprotective effects (6–9, 27–30), which may have influenced the blood levels of syndecan-1. Their anti-inflammatory and endothelial-protective effects might contribute to changes in syndecan-1 levels observed in this study. The implications of our findings extend to the appropriate timing and duration of anticoagulation therapy.

### 4.3 Timing of anticoagulation therapy

In Japan, the J-SSCG 2020 weakly recommends the standard use of heparin as an anticoagulant therapy for sepsis-associated DIC (26). Heparin administration does not improve survival in patients with sepsis and sepsis-associated DIC, nor does it increase the risk of bleeding (31, 32). Evidence suggests that early administration of heparin may improve the prognosis of patients with sepsis. Heparin improves ICU mortality in patients with SIC (33), suggesting that the timing of administration and appropriate therapeutic interventions may indicate the potential efficacy of heparin.

A multinational, randomized, placebo-controlled, double-blind, phase III trial using antithrombin III and rhTM was conducted to investigate their efficacy and safety in adult patients with sepsis and coagulopathy (34, 35); however, the absolute risk of 28-day mortality did not reach statistical significance (34). Despite this, subsequent reports examining the relationship between anticoagulation therapy and mortality in patients with sepsis-associated DIC suggested that mortality may improve (36–38). This discrepancy may be partly due to the lack of uniformity in patient severity; however, the results of this study suggest that the duration of treatment may also be a contributing factor. This study found that anticoagulation therapy improves serum syndecan-1 levels (ameliorates endothelial glycosylation); however, if treatment is discontinued while syndecan-1 levels remain elevated, patients may experience worsening vascular injury and clinical deterioration.

### 4.4 Study limitations

This study has certain limitations. The duration of antithrombin and rhTM therapy varied among the patients. Additionally, we did not assess the association between anticoagulation therapy and adverse events. In this study, all but one case received a combination of antithrombin and rhTM; however, no bleeding events were reported. In addition, the sample size was small, and the data were obtained from a single institution; further large-scale studies are required to confirm these findings.

## 5 Conclusion

Our findings suggest that serum syndecan-1 is a promising biomarker for evaluating vascular endothelial injury and predicting recovery in patients with sepsis-associated disseminated intravascular coagulation (DIC). Patients who exhibited lower syndecan-1 levels after anticoagulant therapy were more likely to recover from DIC, whereas those with persistent elevation of syndecan-1 showed poor outcomes, including mortality. These results indicate that syndecan-1 may be useful for monitoring treatment response and determining the optimal duration of anticoagulant therapy. Incorporating syndecan-1 into clinical decision-making could improve the management of sepsis-associated DIC. Future prospective, large-scale studies are warranted to validate these findings and establish appropriate clinical thresholds.

## Data Availability

The original contributions presented in the study are included in the article/Supplementary material, further inquiries can be directed to the corresponding author.
