# Supplementary material for: Reevaluating anticoagulation therapy for sepsis-associated disseminated intravascular coagulation based on endothelial glycocalyx shedding quantification: a single-center, retrospective, observational pilot study
Source: Front Med (Lausanne). 2025 Jul 16;12:1566753. doi: 10.3389/fmed.2025.1566753 (PMC12307347; doi:10.3389/fmed.2025.1566753)
Supplement: Supplementary file 1 [file Table_1.DOCX]

Supplementary Material

**Supplementary Table 1. Comparative Summary of Diagnostic Criteria for DIC**

| **Criteria** | **Main Features** | **Strengths** | **Limitations** |
| --- | --- | --- | --- |
| ISTH-SIC | Focus on coagulation and organ dysfunction | Simple, useful for sepsis | Less specific for overt DIC |
| ISTH overt DIC | Traditional scoring with platelets, fibrin markers | Gold standard in many settings | May delay diagnosis |
| JAAM-DIC | Includes SIRS, PLT, FDP, PT-INR | Early detection, Japan standard | Some subjectivity in SIRS |
| JAAM-2 | Updated JAAM with revised thresholds | Improved sensitivity and specificity | Still requires external validation |

**Supplementary Table 2. Relationship between recovery from sepsis-associated DIC at the end of anticoagulant therapy and the change in serum syndecan-1 levels after treatment (JAAM-DIC)**

| **Cutoff for**  **syndecan-1 change** | **Sepsis-associated DIC**  **recovery status** | **Syndecan-1 increase ≥ cutoff (n)** | **Syndecan-1 increase < cutoff (n)** | **p-value** |
| --- | --- | --- | --- | --- |
| 10% | Not Recovered (N = 8)  Recovered (N = 5) | 6  1 | 2  4 | p = 0.103 |
| 30% (Main Analysis) | Not Recovered (N = 8)  Recovered (N = 5) | 6  0 | 2  5 | p < 0.05 |
| 50% | Not Recovered (N = 8)  Recovered (N = 5) | 5  0 | 3  5 | p = 0.075 |

Fisher exact test. Since there is no established cutoff value for serum syndecan-1 levels, evaluations were conducted at three points of increase: 10%, 30% (main Analysis), and 50%.
